# Study to increase the pneumococcal vaccination rates of individuals aged 65 years and older

**DOI:** 10.1017/S1463423620000389

**Published:** 2020-09-28

**Authors:** M. V. Biyik, I. Arslan, D. Yengil Taci

**Affiliations:** Health Sciences University, Ankara Training and Research Hospital, Department of Family Medicine, Ankara, Turkey

**Keywords:** pneumococcal vaccination, family medicine, primary care, elderly

## Abstract

**Background:**

In our study, we intended to observe the impact of recommending the pneumococcal vaccine to individuals who were called on the phone or interviewed face-to-face by their doctors on vaccination rates.

**Methods:**

Two hundred individuals who were 65 years old and older were included in our study. They were questioned about their awareness regarding adult immunisation, and their knowledge level and vaccination statuses were determined regarding the tetanus, influenza, hepatitis, and pneumococcal vaccines. After they were given information about the pneumococcal vaccine, they were asked about their interest in being vaccinated. Those who agreed to be vaccinated were invited and vaccinated.

**Results:**

According to the questionnaire, 150 people (75%) knew of the influenza vaccine, 130 people (65%) knew of the tetanus vaccine, 53 people (26.5%) knew of the hepatitis B vaccine, and 49 people (24.5%) knew of the pneumococcal vaccine. A total of five people (2.5%) had received the pneumococcal vaccine. Fifty-eight of 97 patients (59.8%) who completed the questionnaire during a phone call and 84 of 103 patients (81.6%) who completed the questionnaire during a face-to-face interview received the pneumococcal vaccine. As a result, the rates of pneumococcal vaccination increased from 2.5% before the study to 73.5% after the study.

**Conclusion:**

The findings show that the vaccination rates for pneumococcus were very low among our participants. The immunisation rates increased when doctors provided consultation to participants about adult immunisation.

## Introduction

Due to reasons such as increasing numbers of chronic diseases and weakening of the immune system with ageing and environmental factors, increases in the prevalence and severity of infections are observed in older populations. The most cost-effective method for the reduction of mortality, particularly with respect to infections, is primary prevention. As a result of vaccination, a reduction is observed in the frequency and spread of infections. Accordingly, there are decreases in hospitalisation rates, workforce reductions, morbidity and mortality rates, and economic losses (Koldas, 2017; Toprak *et al.*, 2018).

The vaccines that should be administered to individuals who are 65 years of age and older include the seasonal influenza, pneumococcus, tetanus, and hepatitis B vaccines. These vaccines confer protection within 1–2 weeks of administration. These vaccines are provided free of charge for individuals who are aged 65 years and older (Sunay and Demirel, 2011). When necessary, other vaccines (tetanus, diphtheria, pertussis, chickenpox, hepatitis, meningococcus, rabies, typhoid, and cholera) are also recommended for elderly adults based on their level of risk (Hogue and Meador, 2016; Peck *et al.*, 2019).

Pneumococcal disease incidence and mortality increase at the age of 50 years and significantly increase after the age of 65 years. For this reason, it is recommended that individuals aged 65 years and older receive a pneumococcal vaccine. It is also advised that an initial conjugate vaccine (PCV13) and then a polysaccharide vaccine (PPSV23) should be consecutively applied at least 1 year apart, if possible. Booster shot doses are not needed (Ekmud, 2016; Falkenhorst *et al.*, 2017).

The annual rate of pneumococcal vaccination in a vaccination clinic in India was determined to be 7% (Lahariya and Bhardwaj, 2019). Similarly, the rate of vaccination in Turkey is 6.4% (Yılmaz *et al.*, 2018). The immunisation programmes for elderly individuals are still inadequate in Turkey, and despite the availability of effective vaccines, vaccination coverage rates remain low among the elderly population. In our study, we observed the impact of doctor recommendations of the pneumococcal vaccine to individuals on the phone or during face-to-face interviews on vaccination rates.

## Materials and methods

Our study is a cross-sectional study that included 200 patients who were older than 65 years and registered at the Dogantepe Education Family Health Center of the Ankara Training and Research Hospital. Prior to conducting the study, written approval was obtained from the Ethics Board of the Ministry of Health Ankara Training and Research Hospital (22/February/2017 – meeting no. 0675 and no. 5673). The study aimed to include 231 patients who were older than the age of 65 years. Eighteen patients could not be reached due to missing contact information and changes in address. Thirteen patients did not participate in the study. Two hundred patients who were reached and agreed to participate in our study after having been informed about our questionnaire constituted our study sample.

In the questionnaire, which was prepared by the researchers after reviewing the relevant literature, the sociodemographic attributes of the participants (age, sex, educational status, income status, social security status, marital status, number of people living in the house, smoking status, and presence of chronic disease) were collected. The participants were asked if they knew their vaccination status, and if they knew it, they were asked about the source of their knowledge and their knowledge of and attitudes towards the tetanus, influenza, hepatitis, and pneumococcal vaccines.

The questionnaire was administered to 103 patients who attended the polyclinic between 1/March/2017 and 31/May/2017 through face-to-face interviews with their doctors. Ninety-seven patients who did not attend our family health centre were also asked to complete the questionnaires over the telephone. These questionnaires were administered by their family doctors. Following the completion of the questionnaire, the patients were informed about the pneumococcal vaccine and were asked whether they wanted to be vaccinated. The patients who agreed to be vaccinated were prescribed the polysaccharide pneumococcal vaccine at the family health centre.

## Statistical analysis

The statistical evaluation was performed with IBM SPSS 15.0 (SPSS Inc., Chicago, IL, USA). Numerical variables were given as averages ± standard deviations, while categorical variables were given as frequencies (percentages). The relationships between groups were determined with Pearson’s chi-square test and Fisher’s exact chi-square test analyses. Moreover, the factors affecting the dependent variable were determined with logistic regression analysis. In the analyses with multiple variables, *P* < 0.20 was accepted as sufficient for statistical significance in the selection of a covariate. For other sources, *P* < 0.05 was considered to be statistically significant.

## Results

A total of two hundred people who were 65 years of age and older were included in the study. A total of 113 (56.5%) of the participants were women, and the average age was 72.26 years. Regarding education levels, 113 of the participants (56.5%) were literate, while 87 (43.5%) were not. A near-majority of participants (45%) had a monthly income between 1000 and 1499 TL. The average monthly income was 1182 TL. Thirty-eight (19%) of the participants did not receive social security benefits. A total of 66.5% of the participants in the study were married. Statistically significant relationships were not found between pneumococcal vaccination rates and sex, educational status, monthly income, social insurance, marital status and number of people living in the house (*P* > 0.05).

Seventy-three percentage of the participants smoked. The prevalence of chronic disease was 85%. While a statistically significant relationship was not found between smoking status and pneumococcal vaccination rates, the pneumococcal vaccination rates of the patients who had a chronic disease were higher than that of those who did not have a chronic disease (*P* < 0.05). The prevalent chronic diseases were hypertension, diabetes and benign prostatic hyperplasia (Table 1).

**Table 1.** Demographic characteristics

Demographic characteristics (*n* = 200)		*n*	%	*P*
**Sex**	Male	87	43.5	0.24
	Female	113	56.5	
**Educational Status**	Illiterate	87	43.5	0.24
	Literate	33	16.5	
	Elementary school and above	80	40	
**Monthly income (TL)**	0–999	52	26	0.99
	1000–1499	90	45	
	1500 and above	58	29	
**Social security**	Yes	162	81	0.99
	No	38	19	
**Marital status**	Married	133	66.5	0.27
	Single	2	1	
	Divorced	4	2	
	Widowed	61	30.5	
**Number of people living in the house**	1	34	17	0.61
	2	107	53.5	
	3 and above	59	29.5	
**Smoking status**	Yes	15	7.5	0.97
	No	146	73	
	Quit	39	19.5	
**Existence of chronic disease**	Yes	170	85	0.006
	No	30	15	

The knowledge statuses of the participants regarding adult vaccination are shown in Table 2. A total of 86.5% of the participants knew about the vaccines, and more than half of them (59.5%) stated that they had obtained information about the vaccines from doctors or healthcare professionals.

**Table 2.** Awareness of vaccination status among adults and sources of information

Status of knowing adult vaccines and sources of information			*n*	%
**Awareness of vaccination status among adults**		Yes	173	86.5
		No	27	13.5
**Source of information about vaccination**		Doctors or other health professionals	103	59.5
		Pharmacy	8	4.6
		Visual and printed media	20	11.6
		Peers	42	24.3
**Tetanus vaccine**	Does he/she know the tetanus vaccine?	Yes	130	65
		No	70	35
	Has he/she received the tetanus vaccine?	Yes	64	32.0
		No	136	68.0
	Reason for receiving the tetanus vaccine	Animal bite	6	9.4
		Sharp object injury	52	81.2
		Prophylaxis for protection	6	9.4
	Reason for not receiving the tetanus vaccine	Lacking knowledge	130	76.0
		Not believing in vaccination	1	0.6
		Fear	4	2.3
		Negligence	16	9.4
		I did not find it necessary	20	11.7
**Influenza vaccine**	Does he/she know influenza vaccine?	Yes	150	75
		No	50	25
	Does he/she receive influenza vaccine?	Yes regularly	35	17.5
		Yes irregularly	58	29.0
		No	107	53.5
	Reason of not receiving influenza vaccine	Lacking knowledge	56	33.9
		Not believing in vaccination	13	7.9
		Not trusting vaccine	2	1.2
		Fear	5	3.0
		Negligence	62	37.6
		I did not find it necessary	27	16.4
**Hepatitis B vaccine**	Status of knowing hepatitis B vaccine	Yes	53	26.5
		No	147	73.5
	Has the hepatitis B vaccine been applied?	Yes	5	2.5
		No	195	97.5
	Is there any hepatitis B carrier in the family?	Yes	5	2.5
		No	195	97.5
	Reason of not receiving hepatitis B vaccine	Lacking knowledge	153	78.5
		Fear	2	1.0
		Negligence	25	12.8
		Antibody at a protective level	1	0.5
		I did not find it necessary	14	7.2
**Pneumococcal vaccine**	Status of knowing pneumococcus vaccine	Yes	49	24.5
		No	151	75.5
	Has he/she received the pneumococcus vaccine?	Yes	5	2.5
		No	195	97.5
	Reason for receiving pneumococcus vaccine	Lacking knowledge	157	79.7
		Not believing in the vaccine’s efficiency	2	1.0
	Reason for receiving pneumococcus vaccine	Vaccine’s side effects	2	1.0
		Being afraid of the needle	3	1.5
		Negligence	24	12.2
		I don’t find it necessary	8	4.1
		Not being able to access vaccine/expensive	1	0.5

In total, 24.5% (*n* = 49) of the participants stated that they knew about the pneumococcal vaccine. Only 2.5% of the participants had received the pneumococcal vaccine. The predominant reason for not receiving the pneumococcal vaccine was a lack of information (79.7%), and similar results were observed for the other vaccines. It was observed that significantly (*P* < 0.05) more individuals who had chronic diseases than those who did not have chronic diseases had received the pneumococcal vaccine.

**Figure 1.**
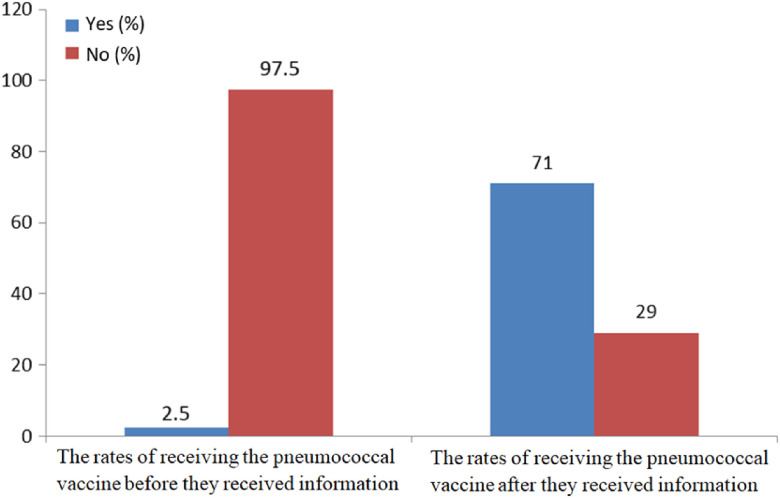
The participants’ rates of receiving the pneumococcal vaccine before and after they received information about it.

After receiving information about the pneumococcal vaccine, 71% (*n* = 142) of the participants agreed to receive the pneumococcal vaccine (Figure 1).

This study was conducted with 200 patients. One hundred three patients (51.5%) attended the polyclinic and participated through a face-to-face interview. Ninety-seven patients (48.5%) who did not attend the polyclinic participated via a telephone call. In total, 58 of the 97 patients (59.8%) who completed the questionnaire via a telephone call and 84 of the 103 patients (81.6%) who completed the questionnaire via a face-to-face interview received the pneumococcal vaccine. The vaccination rate was significantly higher in the group who participated in face-to-face interviews (*P* < 0.001).

The most frequent reasons for refusing to receive the vaccine (35.7%) were finding it unnecessary (35.7%) and worrying about side effects (31%) (Table 3).

**Table 3.** The participants’ reasons of not receiving the pneumococcal vaccine after they received information about it

		***n***	%
**Reasons for refusing to the pneumococcal vaccine after being informed**	Finding it unnecessary	15	35.7
	Worrying about side effects	13	31.0
	Relatives not allowing vaccination	4	9.5
	Not trusting the vaccine efficiency	3	7.1
	The difficulty of accessing the Family Health Center	4	9.5
	Having received the vaccine in the last 5 years	2	4.8
	Lack of insurance coverage or lack of necessary funds	1	2.4
Total		42	100

According to the results of the logistic regression analysis, those without chronic diseases received the pneumococcal vaccine 2.8 times less frequently than those with chronic diseases. Compared to those who did not receive the influenza vaccine regularly, those who did receive the influenza vaccine regularly received the pneumococcal vaccine five times less frequently. Those who received the influenza vaccine irregularly received the pneumococcal vaccine 5.6 times less frequently than those who did not receive the influenza vaccine. The patients who completed the questionnaire during face-to-face interviews received the pneumococcal vaccine 3.2 times more frequently than those who completed the questionnaire over the phone.

## Discussion

The Global Immunization Vision and Strategy of the World Health Organization, ratified by 194 member states in May 2012, was developed to prevent millions of deaths before 2020 by means of increasing access to existing vaccines in all communities. Unfortunately, adult vaccination rates continue to be below the objectives for 2020 for most of the routinely recommended vaccines, in contrast to other preventive services.

In a study that included 2918 patients, it was found that 2.8% received both the pneumococcal and influenza vaccines. The rate of influenza vaccination was 12.3%, whereas 3% of the participants had received the pneumococcal vaccine (Erbay *et al.*, 2018). In our study, 17.5% (*n* = 35) of the participants regularly received the influenza vaccine, 32% (*n* = 64) received the tetanus vaccine, 2.5% received the hepatitis B vaccine and 2.5% received the pneumococcal vaccine. Compared with the data reported in the literature, these data reveal the necessity of exerting more effort to ensure adequate adult immunisation coverage.

At the state level, the “Extended Immunization Program” was initiated to provide immunisation to sensitive age groups to control and even eliminate invasive pneumococcal diseases and other specific diseases. With this system, a consistent 95% vaccination rate was the target among adults (Kim *et al.*, 2018). However, it is difficult for this endeavour alone to achieve this goal. Healthcare professionals and society must also participate. It is important for doctors to focus on consultations and educational services to convey the message to the target audience. In our study, the pneumococcal vaccination rate increased after face-to-face interviews and phone calls.

When the obstacles to adult vaccination are examined, the target audience’s initial vaccination status is not known and patients do not ask to be vaccinated. In addition, patients’ lack of knowledge about vaccines and concerns about the reliability of the vaccines are among the factors that decrease immunisation rates (Hillson *et al.*, 2011; Szilagyi *et al.*, 2005). An experienced family doctor who is willing to vaccinate can overcome this obstacle.

Peetermans *et al.* reported that having a chronic disease is a primary predictive factor for pneumococcal vaccination (Peetermans and Lacante, 1999). In our study, it was found that having a chronic disease affected the pneumococcal vaccination rate. Unfortunately, these rates are not sufficient. Follow-up for chronic diseases should include vaccination reminders.

In the study by Ozisik *et al.*, the lack of knowledge cited as the reason for not receiving the vaccine by 71.4% (*n* = 85) with regard to the pneumococcal vaccine and 64% (*n* = 64) with regard to the influenza vaccine (Ozisik *et al.*, 2016). The failure of healthcare professionals to recommend the vaccine was cited by 21.8% (*n* = 26) with regard to the pneumococcal vaccine and 18% (*n* = 18) with regard to the influenza vaccine. With respect to patients’ knowledge of their pneumococcal vaccination status, 49 of 200 (24.5%) patients participating in our study knew their status. Akman *et al.* found that knowledge of vaccines and vaccination status were very low among the participants (Akman *et al.*, 2014). The results of our study show that adults’ awareness of their vaccination status was poor, which is similar to the findings in other studies. Increasing awareness of vaccines can increase vaccination rates.

When the source of information for the acquired knowledge of adult immunisation was examined, it was observed that knowledge is most frequently obtained from doctors. This was also observed in our study, although the rates in the literature differed from those in our study (Mutlu *et al.*, 2018). In the study by Asik *et al.*, it was stated that 27% of the participants had obtained information about adult vaccines from doctors and/or pharmacists (Asik *et al.*, 2013). In the study by Skowronski *et al.*, this proportion reached 54% (Skowronski *et al.*, 2004). In our study, knowledge was most often obtained from doctors and other health professionals (*n* = 103; 59.5%).

Although the source of knowledge about adult vaccines was mostly doctors or other health professionals, an insufficient proportion of the study population had adequate knowledge of vaccination, given that a lack of knowledge is a critical reason for not receiving vaccinations. This situation indicates that more information about adult vaccines must be given to patients, and the importance of vaccines must be emphasised. In a study by Larson *et al.* (1982), patient education or patient reminders were shown to be effective at increasing community demand for vaccines (Larson *et al.*, 1982). Healthcare professionals should provide more information about vaccines during visits.

In addition, 158 participants (79%) agreed to undergo vaccination after briefly receiving information. While the proportion of those who were vaccinated after a face-to-face interview was 81.6%, it was approximately 59.8% among those who were reached via phone. It is noteworthy that the proportion of vaccinated participants was 2.5% before our study and 71% after our study. The face-to-face interview was found to be 3.2 times more effective than the phone call. These results demonstrate the importance of providing consultation regarding vaccination to improve vaccination rates. Moreover, the contribution of the patient–doctor meeting is undeniably important.

Adult vaccination plays an important role in preventive medicine, is easily applicable and is efficient and cost-effective. Although chronically ill patients visit their doctors frequently, it is evident that opportunities for vaccination had been missed in this group. Missed opportunities for vaccination in elderly adults represent a very important problem worldwide (Nowalk *et al.*, 2005). Our study revealed that it is necessary to pay more attention to the administration of vaccinations, and doctors have important responsibilities with regard to increasing vaccination rates.
